# Global Health Education during the COVID-19 Pandemic: Challenges, Adaptations, and Lessons Learned

**DOI:** 10.4269/ajtmh.21-0773

**Published:** 2021-10-11

**Authors:** Kristina M. Krohn, Michael A. Sundberg, Nasreen S. Quadri, William M. Stauffer, Adriana Dhawan, Hope Pogemiller, Viviane Tchonang Leuche, Sarah Kesler, Tsige H. Gebreslasse, Megan K. Shaughnessy, Bobbi Pritt, Alma Habib, Beth Scudder, Sarah Sponsler, Stephen Dunlop, Brett Hendel-Paterson

**Affiliations:** ^1^ Global Medicine, Department of Medicine, University of Minnesota Medical School, Minneapolis, Minnesota;; ^2^ Department of Pediatrics, University of Minnesota Medical School, Minneapolis, Minnesota;; ^3^ Internal Medicine, Allina Health, Minneapolis, Minnesota;; ^4^ Internal Medicine, Hennepin Healthcare, Minneapolis, Minnesota;; ^5^ Internal Medicine HealthPartners, St. Paul, Minnesota;; ^6^ Department of Laboratory Medicine and Pathology, Mayo Clinic, Rochester, Minnesota;; ^7^ Emergency Medicine, Hennepin Healthcare, Minneapolis, Minnesota

## Abstract

Global health education programs should strive continually to improve the quality of education, increase access, create communities that foster excellence in global health practices, and ensure sustainability. The COVID-19 pandemic forced the University of Minnesota’s extensive global health education programs, which includes a decade of hybrid online and in-person programing, to move completely online. We share our experience, a working framework for evaluating global health educational programming, and lessons learned. Over the decades we have moved from a predominantly passive, lecture-based, in-person course to a hybrid online (passive) course with an intensive hands-on 2-week requirement. The pandemic forced us to explore new active online learning models. We retained our on-demand, online passive didactics, which used experts’ time efficiently and was widely accessible and well received. In addition, we developed a highly effective synchronous online component that we felt replaced some of the hands-on activities effectively and led us to develop new and innovative “hands-on” experiences. This new, fully online model combining quality asynchronous and synchronous learning provided many unanticipated advantages, such as increasing access while decreasing our carbon footprint dramatically. By sharing our experience, lessons learned, and resources, we hope to inspire other programs likewise to innovate to improve quality, access, community, and sustainability in global health, especially if these innovations can help decrease negative aspects of global health education such as its environmental impact.

In 2020, global health education programs, which historically have been highly dependent on international travel, were forced to adapt to sudden restrictions as a result of COVID-19. Responses ranged from halting or limiting activities to transitioning quickly to online platforms.^1^ As the pandemic changes and programs move forward, it is critical to consider whether some of the adaptations hold valuable lessons for improving the future of global health education. To stimulate widespread programmatic evaluation, we describe our experience adapting the University of Minnesota’s extensive global health education program, including our American Society of Tropical Medicine and Hygiene (ASTMH)–accredited Global Health Course, to accommodate COVID-19 restrictions. We describe both our challenges and successes as we seek to improve educational quality, accessibility, and foster community while simultaneously decreasing our environmental impact and ensuring better use of resources. We hope these reflections will be useful for other global health programs, and will stimulate further sharing and dialogue.

## PRE-PANDEMIC: COURSE BEGINNINGS AND EVOLUTION

The University of Minnesota Global Health Course was launched in 2005, consisting of 8- to 9-hour days of mostly didactic (passive) lectures, with interspersed laboratory time over 8 weeks. Recognizing the need for increased flexibility for our diverse learners, many of whom were practicing clinicians, the course transitioned to a hybrid online/in-person format in 2010. Participants watched online asynchronous didactics in advance of the course to obtain essential knowledge for full participation in the 2-week intensive hands-on course component. Although the information in our courses is rated by learners to be excellent and contains all the necessary material to prepare individuals to sit for the ASTMH CTropMed^®^ examination or a diploma in tropical medicine and hygiene through the Royal College of Physicians, the format of mostly passive lectures left room for improvement. Passive learning is inferior to active methods that have emerged as best practices of medical education.^2^^–^^5^ Like many courses, our course directors have been limited by lack of time and funding for investigating and using new instructional technologies.

Hosting the in-person component in Minnesota remained an obstacle for international participants. We partnered with Makerere University in Kampala, Uganda, and Mahidol University in Bangkok, Thailand, to improve quality and access. Learners would take our online content and use time in Thailand or Uganda for the hands-on component. Using different locations focused content on a given locale’s diseases, cultures, needs, and resources, thereby providing high-quality immersive experiences. Subsidizing local and regional students increased access for learners from low- and middle-income countries (LMICs). The courses are co-created and taught by in-country expert colleagues, so our learners benefit from their experience and expertise practicing tropical medicine and global health. This approach also decreased faculty travel costs and improved representation of perspectives from colleagues in the “Global South.”^6^ Regardless of location, our intensive in-person courses create a sense of community, camaraderie, and collegiality that last long past the course itself. The online sphere was different. Although we established discussion boards and e-mail listservs, we struggled to create a sense of connection pre-pandemic.

In-person training either requires travel or excludes some individuals based on geography and is ultimately detrimental to our planet’s sustainability. The cost and carbon emissions of long-distance travel for experts and course participants of international conferences is recognized increasingly, as demonstrated by analyses of the ASTMH and Radiological Society of North America annual meetings.^7^^,^^8^ Additionally concerning is the fact that the negative effects of climate change affect LMICs disproportionately—the very populations we hope ultimately benefit from our courses.^9^^,^^10^

## COVID-19: A FORCED OPPORTUNITY WITH A STEEP LEARNING CURVE

As all in-person training and travel ceased in March 2020, we questioned the immediate and long-term viability of our courses. For years pre-pandemic, we strived to improve quality, access, community, and sustainability. COVID-19 threatened to remove the improvements we had achieved and highlighted our shortcomings. Although our transition to fully online content delivery was borne out of necessity, it provided an opportunity to make large-scale improvements to our program. Here we describe how our adaptations succeeded or failed based on the values of improving quality, facilitating accessibility, fostering community, and sustainability.

### Maintaining and expanding high-quality online education.

The pandemic accelerated our shift to and appreciation of active online learning. Having hired an academic technologist and trained faculty in andragogy and online education theories helped us create new promising practices. Small-group case discussions, such as the “pre- and post-travel case” provided in Supplement 1, worked well online, especially as the pandemic progressed and participants became more familiar with engaging in online video groups. Small-group online tabletop exercises and virtual simulations created a sense of community among learners, and taught important skills through team decision making and problem-based learning, which follows best practices of adult education.^3^^–^^5^ A sample virtual simulation facilitator guide and slides are shared in Supplements 2 and 3. We conducted virtual laboratories using digital microscopy paired with an interactive question–answer format. During these live sessions, the instructor projected the microscope field onto a computer screen using a digital camera and associated software. The learners viewed the instructor’s screen in real time and watched as microorganisms of interest were located and analyzed. We felt this offered an improved experience over our conventional laboratory sessions during which learners rotated among microscope sessions with pre-configured displays, because the learners could now observe the process of identifying the various microorganisms. On the other hand, the virtual experience did not allow the learners the opportunity to “drive” the microscope and attempt to find the microorganisms themselves, which is another component of our in-person hands-on laboratories.

We paired these live components with asynchronous content. Asynchronous content is similar to homework; it is learning done outside of class time with teachers. We specifically used recorded lectures, readings, and interactive modules that provide high-quality content in multimedia formats that combine shorter videos, readings, and questions. An example of an interactive asynchronous module, “Cancer in Low-Resource Settings,” showcasing one of our local colleagues in Tanzania, is shared in Supplement 4. Although these activities were well received, they required significant preparation, expertise, and time to create, making them demanding for our faculty.

When comparing the quality of our synchronous online sessions with our previous in-person course, our learner evaluations remained stable. Participants are asked each day to rank the days materials as excellent, very good, good, fair, or poor. In 2018, 2019, and 2021, the mode for each day with data was very good or excellent, with 12 “excellent” and seven “very good” in 2018, 13 “excellent” and five “very good” in 2019, and 17 “excellent” and three “very good” in 2021. In 2020, 2 days received a mode evaluation of “good”, one “very good”, and 16 “excellent.” The final exam for this portion of our course is created from a question using many of the same multiple-choice questions year to year. The average scores in 2017 and 2019 when in-person was 69% (SD, 21; *n* = 139). Final scores on the exam from 2018 were not saved. In 2020, we did not offer a final exam. In 2021, during the completely online course, the average on the final exam was 79% (SD, 17; *n* = 48). As a CTropMed Diploma Course, the ultimate test will be the quality of care provided by our learners and pass rates for the CTropMed exam. At the time of this writing, the CTropMed exam has not yet occurred for the learner cohorts who took our online courses, and clear quality alternative methods for evaluating medical e-learning have yet to be determined.^11^^–^^14^

### Expanding access.

Some attempts at asynchronous learning pre-pandemic grew exponentially during the pandemic as institutions around the world sought virtual education, particularly content like ours, which focuses on better health care for underserved populations. The WHO is translating our dermatology course into French, Spanish, and Portuguese to be disseminated through OpenWHO to low-resource settings later in 2021. The University of Minnesota, like many other universities, removed all students from clinical activities early during the COVID-19 pandemic to save personal protective equipment for fully trained personnel and to protect learners. As a result, the medical school, like many medical schools around the world, rapidly sought elective virtual curricula.^15^^–^^23^ When the university asked for virtual electives, we as faculty in global medicine rapidly converted our online curriculum designed for continuing medical education to instruct more than 120 medical students on immigrants and refugee health, global health, and tropical medicine. In-person courses could never have been expanded in these ways. As students have returned to in-person clinical settings to complete their required clinical rotations, we have maintained some of the online elective options. Students continue to choose the virtual electives that help them learn to care better for our diverse communities, with 50 enrollments in our electives in the first half of the 2021–2022 schoolyear.

Virtual learning spaces removed the barriers of distance and travel. Colleagues could join and provide their perspectives from across the United States, and countries including Panama, Thailand, Jordan, the United Kingdom, Australia, New Zealand, Gabon, Kenya, and Tunisia. Often, the people best suited to teach were deeply involved in clinical or public health interventions related directly to the pandemic and did not have time to travel, but could attend a video meeting. Sometimes experts involved in COVID-19 responses could not even meet during typical class times, but did have time to record a lecture or share a personal story. We scheduled 2 synchronous hours and 2 asynchronous hours per day for 4 weeks rather than our traditional 2 weeks of 8-hour days. Participants appreciated the flexibility, the shortened days, and the longer duration, as many said they would not have been able to take the time off to attend a full-day, week-long course, but could adjust clinic schedules for a few hours each day over the month. We recorded most of the online discussions and were able to select, edit, and share high-yield recordings with those who could not attend synchronously. It was a good reminder that passive learning may be appropriate depending on the context and content, in particular when it increases access.

Difficulties with Internet bandwidth and time zones prevented some participants’ full participation. Most sessions in 2020 and in 2021 had attendance rates of more than 80%, with all students in 2021 being able to attend a minimum of 15 of the 20 synchronous days. We do not know how many people chose not to participate because of time zone constraints. Interactive video sessions required greater bandwidth and excluded some participants, although some adaptations could be made by using audio only. Other participants were limited by time zone differences. Pre-recorded sessions required lower bandwidth, were more accessible, and were valued by course participants in lower resource settings who may otherwise have been excluded from participating in our course.

We noted that the online environment allowed greater geographic representation in both synchronous and asynchronous activities, leading to broader representation of colleagues from the Global South as educators. Time, travel, and financial barriers may have made their participation in a U.S.-based course more difficult. In one example, colleagues at partner sites in Laos, Tanzania, India, and the United States participated in a very well-received synchronous panel discussion. International instructors also gave lectures over Zoom. The “digital divide” remained, however, and at times we struggled to accommodate slow Internet connections. We adapted by having instructors forward slides for us to display, and by accessing the video sessions through voice-only options when needed.

One value that we have not and cannot adequately address in this article is the desire to be more equitable in our global health programs. To be more equitable, we must address the colonial history of global health and tropical medicine, and we must acknowledge the ways in which many of our current models perpetuate inequities. Evaluating educational interventions based on equity requires defining who the audience is and who has the right to determine what is considered equitable. Moving online is insufficient to make global health education equitable, and it is beyond the scope of this article to discuss true equity. Therefore, we focused on improving access. We have offered significant discounts to our courses for participants from LMICs, waived fees and supported learners from our partner sites, and sponsored travel to Minnesota for LMIC colleagues to attend our course. Moving fully online could potentially decrease costs further for participants—both the cost of the course and removing the cost of travel, which can increase access dramatically. For our usual in-person course in pre-COVID years, we averaged spending around $10,000 on travel for speakers. This does not include waiving the course fee and supporting travel and accommodations to make it possible for learners from LMICs to attend the course. Because we had invested previously in hiring an academic technologist for our courses, the University of Minnesota already had a Zoom contract, and we had previously dedicated faculty time to online education, these costs remained fixed for 2020 and 2021.

### Fostering community.

One of our biggest fears in moving our Global Health Course fully online was the loss of community. However, seeing our colleagues in their homes, with their families coming in and out of the screen, added a new dimension to our lives. Virtual meetings forced many of us to share our private lives with our colleagues in different, often beneficial, ways. Many of us who previously kept work and family separate now bonded over the shenanigans of children or pets vying for more attention. Leaving Zoom meetings open after class time officially ended, and having open break-out rooms that participants could use for more individual conversations, and side bar chats all helped encourage some of the side conversations that happen in person. These interactions do not replace completely hallway conversations or the discussions shared during meals; however, we were surprised by the depth of community we were able to create.

Another pleasant surprise was the ability to create some community online through small and large groups. We kept our class size to less than 50 individuals to ensure that participants could ask questions and start discussions with the experts. Participants and experts from around the world were able to share the similarities and differences in their experiences in real time, and a number of our participants bonded and planned to collaborate on future endeavors.

### Sustainability and environmental responsibility.

Lack of global or domestic travel to our courses led to a much lower environmental impact. For our in-person course in 2019, there were 38 traveling participants—one international speaker, 10 domestic speakers, nine international participants, and 18 domestic participants from outside of Minnesota—compared with zero traveling participants in 2020 and 2021. Being online eliminated travel and decreased time demands on experts in the United States and from around the world, which was particularly important for updates related to ongoing COVID-19 outbreaks in different locales. Our asynchronous online content is enduring material, which can be improved iteratively over time. Our synchronous “live” sessions, although more time- and faculty-intensive to create, will serve as a base for leading similar sessions in the future.

## MOVING INTO THE FUTURE

We acknowledge that online education is different from in-person learning, and there is still great disparity in online education as a result of the digital divide, particularly for learners without high-speed Internet access. We found new challenges were introduced by accounting for learners in multiple time zones when planning live virtual activities. We are now critically analyzing the benefits, drawbacks, challenges, and solutions for improving online global health education. We summarize these in Tables 1 and 2 in the hopes others can learn from our experience.

**Table 1:** Benefits and drawbacks of online education in global health as identified through discussions and experience of University of Minnesota Global Health and Tropical Medicine Curriculum faculty, 2020 through 2021

Benefits of online education	Drawbacks of online education
Technology investments result in novel teaching methods.	There is a lack of an in-person community.
Allows learners to select education courses from around the world, not just in their physical location.	It is exclusionary based on Internet bandwidth.
Learners can customize their education.	It is exclusionary based on time zones.
Learning can be self-paced for individual learning styles and can accommodate those with busy schedules.	Some benefits are lost from in-person, hands-on mentored experiences (physical skills in the laboratory and procedures).
Flexible asynchronous activities can increase access for diverse participants significantly.	The creation of interactive materials is intensive in terms of time and technology.
One is able to increase involvement from international/remote faculty in live virtual sessions.	Assessing the quality of education is difficult until after significant amount of time and investment.
Material may be reused and shared easily and broadly when appropriate.	Up-front investment and different skill sets are required to create quality online learning.
There is the potential to decrease costs for participants.	Assessing competence requires different techniques.
There is decreased environmental impact.	

**Table 2:** Challenges for the future of online global health education, with potential solutions as identified through discussions and experience of the University of Minnesota Global Health and Tropical Medicine Curriculum faculty, 2020 through 2021

Challenges for the future	Proposed solutions
Faculty/participants may be inexperienced with technologies or learning modalities.	Have dedicated staff or faculty prep experts for different technologies used. Conduct practice run-throughs before live sessions, with an eye on video and audio quality.
Asynchronous interactive modules take time and investment to create.	Invest in training staff and faculty in creating quality interactive online content.
Need variety to prevent “Zoom fatigue.”	Invest in instructional design to improve usability and variety for participants.
Manage multiple time zones.	Select timing carefully to ensure synchronous content.
A “digital divide” limits access for participants/partner faculty with low bandwidth.	Invest in digital infrastructure for partners. Support worldwide Internet access. Share slides before sessions; include audio-only access.
Improve worldwide representation of experts.	Invest and train staff and faculty at partner sites in creating quality online education. Create templates and clear instructions for experts, highlighted in online materials, to make participation as easy as possible.

Although the pandemic has taken, and continues to take, a terrible toll, its disruption has forced innovation in global health education and galvanized investment in online education methodologies. We have learned that investing time, energy, and people has the potential to increase flexible access dramatically to quality online education and can better meet best practices for adult education while decreasing our carbon footprint. At the University of Minnesota, we are exploring new hybrid and fully virtual course models. We are seeking regional collaborations for hands-on courses that build local capacity by highlighting local faculty, that improve access through local scholarships, and that increase community and mutual benefit through matching students from high-income countries with students from LMICs. In addition, we are investing time and energy into both innovative active online learning and lower bandwidth online options to improve quality and access further for all our colleagues. We hope to collaborate with ASTMH-accredited courses and others to create, share, and disseminate quality teaching materials as the online materials are easier to share among institutions. We hope our experience is helpful to all global health educators who are critically evaluating their own programs with the intent of maximizing quality and access for all, thereby supporting those who seek to learn, teach, and practice global medicine in ways that promote sustainable global community.

## Supplemental Material


Supplemental materials
