# Supplementary material for: Global Health Education during the COVID-19 Pandemic: Challenges, Adaptations, and Lessons Learned
Source: Am J Trop Med Hyg. 2021 Oct 11;105(6):1463–7. doi: 10.4269/ajtmh.21-0773 (PMC8641357; doi:10.4269/ajtmh.21-0773)
Supplement: Supplementary file 1 [file tpmd210773.SD1.pdf]

Supplement 3: Interactive asynchronous module on Cancer in Low Resource Settings is accessible here: https://rise.articulate.com/share/LpG0Dg0G8vcRqP_StDxw28zQOnswhlz-#/

Travel Medicine Session Participant Handout: Pre-Travel

Please complete the online activity "An Introduction to Travel Medicine." Next, review the following scenarios in advance to the pre-travel cases group session. Resources include the CDC website (www.cdc.gov/travel) and Global TravEpiNet (http://gten.travel/prep/prep)

====== SCENARIO =============

Traveler: 22-year-old woman (US born), recently graduated from college.
Past Medical History: ADHD
Medications: Adderall. No drug allergies.
Vaccination history: Has completed all childhood immunizations (DTaP, Hep B, IPV, Hib, Prevnar, MMR, Varicella, Meningococcal, HPV). Has a record with only 1 hepatitis A vaccine, administered at age 12. Last tetanus shot was Tdap 1 year ago. Already received a flu shot this year.

Trip: Taking a 3-month trip to SE Asia (leaving in 6 weeks). Self-arranging her trip and the itinerary is flexible. Flying into Bangkok, Thailand. Plans to spend 4-6 weeks traveling around Thailand, then flying to Siem Reap, Cambodia. Will spend an unknown amount of time in this area. Next flight will be to Hanoi Vietnam for 1 week, then a Halong Bay cruise for 5 nights followed by a trip down the east coast of Vietnam visiting beach towns for 2 weeks. Hopes to go to Ho Chi Minh City then to some South Vietnam villages in the Mekong Delta, then home. She plans to do some rafting and possibly swim in the Mekong river.

Pre travel counseling:
Vaccines:

Medications:

- Malaria (if needed):

- Travelers Diarrhea (if needed):

Special Considerations/Counseling:

** What if she was spending a lot of time along the borders of Thailand?

** What if she was leaving in 2 weeks instead of 6 weeks?

Travel Medicine Session Faculty Handout: Pre travel

Learning Objectives:

At the end of this session, participants should be able to:

- Describe key components of a consult for pre-travel preparation
- Recognize characteristics of unique traveler populations (such as the VFR traveler, the pregnant traveler, the immunocompromised traveler)
- Describe unique areas for focus of counseling for specific travel populations and travel destinations

Structure and Facilitator Guide/Notes:

- THANK YOU!!! We are grateful for your expertise and generosity of time.
- The session begins at 10am. We will begin together and divide the participants into small groups in Zoom "Breakout Rooms." The course administrators will direct you to your zoom room. Expect to have about 25min for each scenario. You will repeat the scenario 4 times with a new group each time. The course administrators will move you from group to group.
- Prior to the session, class participants should have watched the Introduction to Travel Medicine video, worked through the example pre travel scenario, and reviewed the four scenarios provided in advance (Case 5 will only be discussed if extra time during a session).We are expecting them to use TravEpiNet PREP (https://gten.travel/prep/prep) and/or the CDC travel page to answer the questions
- Please be patient and flexible - this is a new format for us. We are learning a lot as we go.
- Our course involves a variety of learners with a variety of experience and backgrounds (medical students to retired physicians, family medicine, emergency medicine, medicine, pediatrics). We will do our best to mix up small groups, but it is a difficult task.
- The learner handouts are attached at the end of the document. They are essentially the faculty handouts but without the **Bolded** information.
- We expect that most of the issues in bold are things that you will have seen in a travel clinic. If you have questions about your assigned case, we suggest looking up the **Focus** for the case in the Yellow Book or email the faculty lead for this session.
- **Please take note of the areas of focus for your case. This is an attempt to diversify the case discussions (so not every faculty lead talks about Rabies vaccine while no one talks about JE, for example) and highlight important points unique to your case. These are again included in the special considerations section and underlined when relevant in the vaccines/medications section.**
- Try to have the learners go through the case and answer the questions
- If you finish early, please feel free to lead the discussion around travel medicine topics that you feel are related or interesting.
- In addition to your assigned case (Case 1-4), we have included a Case #5 - of a Hajj traveler with hypertension and diabetes. If you find that for some reason your discussion of your topic went very quickly and you have time left over, feel free to start going through this case.
- **Of note, we have not included a specific discussion about COVID-19 counseling and testing requirements for each case, as this changes regularly for individual countries. However, anticipate that this may be a question brought up by learners, that we could discuss together as a larger group prior to or after breakout sessions.**

====== SCENARIO =============

FACULTY LEAD: ***

Areas of Focus: Malaria chemoprophylaxis options when traveling in and out of malaria areas, Japanese encephalitis vaccination, Hep A series (2 doses, don't need to restart if long interval in between), STDs/high risk activities, safety concerns, traveling with controlled medications, Antimalarial drug resistance rates in SE Asia

Traveler: 22-year-old woman (US born), recently graduated from college
Past Medical History: ADHD
Medications: Adderall. No drug allergies
Vaccination history: Has completed all childhood immunizations (DTaP, Hep B, IPV, Hib, Prevnar, MMR, Varicella, Meningococcal, HPV). Has a record of only 1 hepatitis A vaccine, administered at age 12. Last tetanus shot was Tdap 1 year ago. Already received a flu shot this year.
Trip: Taking a 3-month trip to SE Asia (leaving in 6 weeks). Self-arranging her trip and the itinerary is flexible. Flying into Bangkok, Thailand. Plans to spend 4-6 weeks traveling around Thailand, then flying to Siem Reap, Cambodia. Will spend an unknown amount of time in this area. Next flight will be to Hanoi Vietnam for 1 week, then a Halong Bay cruise for 5 nights followed by a trip down the east coast of Vietnam visiting beach towns for 2 weeks. Hopes to go to Ho Chi Minh City then to some South Vietnam villages in the Mekong Delta, then home. She plans to do some rafting and possibly swim in the Mekong river.

<u>Pre travel counseling:</u>
Special Considerations/Counseling:
-STDs: safe sex practices and getting tested for STDs after return
-Safety/security: young woman traveling alone
-Freshwater swimming: doxycycline for leptospirosis prophylaxis (200 mg weekly)?, Schistosomiasis
-Controlled medications (including ADHD meds) require medication letters and/or may not be allowed into certain countries.

Vaccines: ***<u>Hep A (completes series)</u>, Typhoid, <u>Japanese Encephalitis</u>. Discuss/consider rabies pre-exposure vaccination series.***

Medications:

- Malaria: ***malarone or doxycycline. <u>Mefloquine?</u> Concern for drug resistance along the borders of Thailand and her itinerary is flexible, so not the best choice.***
    - ***<u>Prophylaxis for the entire trip or just risk locations? Requires discussion with the traveler and only a reasonable choice if using malarone.</u>***
- Travelers Diarrhea: ***Azithromycin, <u>no ciprofloxacin due to drug resistance concerns.</u>***

** What if she was spending a lot of time along the borders of Thailand? ***Mefloquine resistance***

** What if she was leaving in 2 weeks? ***Accelerated series for JE at 0 and 7 days instead of 0 and 28 days, not enough time for 3 dose rabies series.***

Travel Medicine Session Participant Handout: Pre-Travel

Please complete the online activity "The Ill Returned Traveler." Next, review these scenarios in advance to the online group session. You may recognize the travelers from the pre-travel day. We have included "answers" to their pre-travel preparation. Look at the post-travel cases below and use any available resources. We suggest the CDC travel website (www.cdc.gov/travel) or the CDC yellow book https://wwwnc.cdc.gov/travel/page/yellowbook-home-2014 - particularly the Post-travel evaluation under "Clinician Tools and Resources" or look for post-travel topics in the CDC yellow book table of contents.

====== SCENARIO =============

Traveler: Healthy 22-year-old woman, recently graduated from college
Trip: Taking a 3-month trip to SE Asia (leaving in 6 weeks). Self-arranging her trip and the itinerary is flexible. Flying into Bangkok, Thailand. Plans to spend 4-6 weeks traveling around Thailand, then flying to Siem Reap, Cambodia. Will spend an unknown amount of time in this area. Next flight will be to Hanoi Vietnam for 1 week, then a Halong Bay cruise for 5 nights followed by a trip down the east coast of Vietnam visiting beach towns for 2 weeks. Hopes to go to Ho Chi Minh City then to some South Vietnam villages in the Mekong Delta, then home. She plans to do some rafting and possibly swim in the Mekong river.

Post-Travel:
HPI: Fever started the day before she got on the plane to return to the US and has continued for the past 3 days. Has a headache, malaise, fatigue, mild nausea. No cough, no diarrhea, no jaundice, no rash. Reports frequent insect bites throughout her trip, although did use insect repellent. Drank bottled water but ate fresh fruits/veggies, had raw sushi, and frequently ate food from street vendors. Rode elephants and helped bathe them in stagnant ponds. Went rafting with swimming in a freshwater river. Reports two new sexual partners with variable condom use.

Exam: Temp 102.5 Pulse 110 Blood pressure 110/55 Respiratory rate 12 Oxygen sat 99%
Gen: Fatigued appearing but in no acute distress
HEENT: No icterus, no oral lesions
Lungs/CV normal except for tachycardia
Abdomen: No Hepatosplenomegaly
Musculoskeletal: no joint pain/swelling
Neuro: Alert and answering questions appropriately. Normal muscle tone and strength.
Skin: no rashes

- What is your differential?
- What are your priority tests (i.e. what will you order stat)?
- What other tests/labs would you want? (your faculty will give you the values if available)
- How would your differential change if the fevers began 4 weeks after returning home?

Travel Medicine Session Faculty Handout: Post travel

Learning Objectives:
At the end of this session, participants should be able to:

- Describe initial work-up and management of the ill returning traveler

Structure and Facilitator Guide/Notes:

- THANK YOU!!! We are grateful for your expertise and generosity of time.
- The session begins at 10am. We will begin together and divide the participants into small groups in Zoom "Breakout Rooms." The course administrators will direct you to your zoom room. Expect to have about 25min for each scenario. You will repeat the scenario 4 times with a new group each time. The course administrators will move you from group to group.
- Prior to the session, class participants should have watched the Ill returned Traveler lecture and reviewed the four scenarios provided in advance (Case 5 will only be discussed if extra time during a session).We are expecting them to use the CDC travel website (www.cdc.gov/travel), or the yellow book (https://wwwnc.cdc.gov/travel/yellowbook/2020/table-of-contents)
- Please be patient and flexible - this is a new format for us. We are learning a lot as we go.
- Our course involves a variety of learners with a variety of experience and backgrounds (medical students to retired physicians, family medicine, emergency medicine, medicine, pediatrics). We will do our best to mix up small groups, but it is a difficult task.
- The learner handouts are attached at the end of the document. They are essentially the faculty handouts but without the **Bolded** information.
- We expect that most of the issues in bold are things that you will have seen in your practice. If you have questions about your assigned case, we suggest looking up the **Focus** for the case in the Yellow Book or email the faculty lead for the session.
- Try to have the learners go through the case and answer the questions.
- If they ask for a lab that is not listed, feel free to either make one up, say we don't have it, or say that it doesn't matter.
- If you finish early, please feel free to lead the discussion around travel medicine topics that you feel are related or interesting.
- In addition to your assigned case (Case 1-4), we have included a Case #5 - of a Hajj traveler returning with a respiratory infection. If you find that your discussion of your topic went very quickly and you have time left over, feel free to start going through this case.

====== SCENARIO =============
FACULTY LEAD:

Traveler: Healthy 22-year-old woman, recently graduated from college
Trip: Taking a 3-month trip to SE Asia (leaving in 6 weeks). Self-arranging her trip and the itinerary is flexible. Flying into Bangkok, Thailand. Plans to spend 4-6 weeks traveling around Thailand, then flying to Siem Reap, Cambodia. Will spend an unknown amount of time in this area. Next flight will be to Hanoi Vietnam for 1 week, then a Halong Bay cruise for 5 nights followed by a trip down the east coast of Vietnam visiting beach towns for 2 weeks. Hopes to go to Ho Chi Minh City then to some South Vietnam villages in the Mekong Delta, then home. She plans to do some rafting and possibly swim in the Mekong river.

<u>Post-Travel</u>: Fever, early onset. **Diagnosis: Dengue**
HPI: Fever started the day before she got on the plane to return to the US and has continued for the past 3 days. Has a headache, malaise, fatigue, mild nausea. No cough, no diarrhea, no jaundice, no rash. Reports frequent insect bites throughout her trip, although did use insect repellent. Drank bottled water but ate fresh fruits/veggies, had raw sushi, and frequently ate food from street vendors. Rode elephants and helped bathe them in stagnant ponds. Went rafting with swimming in a freshwater river. Reports two new sexual partners with variable condom use.

Exam: Temp 102.5 Pulse 110 Blood pressure 110/55 Respiratory rate 12 Oxygen sat 99%
Gen: Fatigued appearing but in no acute distress
HEENT: No icterus, no oral lesions
Lungs/CV normal except for tachycardia
Abdomen: No Hepatosplenomegaly
Musculoskeletal: no joint pain/swelling
Neuro: Alert and answering questions appropriately. Normal muscle tone and strength.
Skin: no rashes

(Recommend referencing CDC Yellow Book Tables 11-01 and 11-03)

- What is your differential? ***Malaria, Dengue, Chikungunya, Zika, typhoid fever, Leptospirosis, Rickettsial diseases, Melioidosis, STDs including acute HIV, Influenza, Japanese Encephalitis, COVID, Schistosomiasis, others?***
- What are your priority tests (i.e. what will you order stat)? ***Malaria rapid diagnostic test and thick/thin smear, complete blood count with differential (CBC/diff), blood cultures***
- What other tests/labs would you want? ***Basic metabolic panel (BMP), liver function tests, urinalysis (UA), Influenza, COVID PCR, respiratory virus panel, chest Xray, HIV***
  Lab results:
  BMP normal.
  Total bilirubin normal, Aspartate aminotransferase (AST) 90, Alanine aminotransferase (ALT) 101
  UA normal
  Influenza, COVID, and Respiratory virus panel negative. Chest Xray is normal.
  HIV negative (viral load and combo test)
  Malaria negative (rapid diagnostic test and smear)
  White blood cell count 3.2 (normal differential), Hemoglobin 13.7, platelets 72
  Blood cultures no growth to date

  How does the differential now change based on the lab results?
  No HIV, unlikely to be malaria or respiratory virus
  Would expect higher bilirubin if leptospirosis
  Thrombocytopenia increases likelihood of d, Dengue, Chikungunya, Zika, Rickettsial diseases

- How would your differential change if the fevers began 4 weeks after returning home? ***No Dengue, Chikungunya, Zika, leptospirosis, Rickettsial diseases or Japanese Encephalitis based on the shorter incubation periods***

Learning Objectives:

1) Recognize the presentation of a viral hemorrhagic fever such as Ebola virus disease (EVD) and institute appropriate isolation and infection prevention procedures.
2) Initiate appropriate resuscitation of critically ill patient and patient with suspected EVD.
3) Initiate management of disease outbreak in low-resource countries (communication and collaboration, case management, infection prevention and control practices, surveillance and contact tracing, safe lab practices, safe and dignified burials, and social mobilization).
4) Recognize emerging therapies for EVD.
5) Recognize the need for EVD survivor medical management and social support after recovery.

Facilitator Instructions for virtual sim (zoom platform):

The facilitator and a small group of participants (ideally 7 or less people) will join a zoom call together for this simulation. The facilitator follows the steps for this simulation as outlined below, sharing their screen to show the slideshow and advancing slides as indicated as the case progresses. If there are pauses, the facilitator asks the team what they would like to do next. After 20 minutes, the scenario and slideshow end, and the facilitator can use the EVD handout at the end of these instructions to debrief.

Introduction:

Slide 1: Virtual Simulation

The facilitator asks everyone to introduce themselves and then give the following information

You will have approximately 20 minutes for this scenario. During this encounter, you should:

- Assess the patient and situation by taking a history and asking for vital signs and exam findings.
- Plan appropriate responses and verbalize all of your actions.
- Prioritize your care based on the situation and patient status.
- Verbalize your interventions to the faculty for the simulation.

Remember to use key elements that form the basis of care with any patient, including hand hygiene, patient and provider safety, teamwork, and delegation. The facilitator for the case will play roles of people in the scenario as well as guiding you through the scenario. The facilitator will notify you when the scenario is at a close and will lead a debrief at the end.

about this virtual simulation:

Click “next” to advance the slide. Slide 2 will show.

Setting the Scene:

Slide 2: House in a rural village near Makeni, Sierra Leone.

Facilitator asks one participant to read “The simulation case,” below to set the scene with the location and hospital capabilities.

The simulation case:
You are working in a small mission hospital in rural Sierra Leone, near Makeni. The closest hospital with a higher level of care is 50 km away. This hospital receives referrals from many of the very small health clinics/hospitals in the surrounding areas. It also receives walk-in patients at all hours. A family member comes in with a patient, a 24 year-old male.

Click “next” to advance the slide. Slide 3 will show.

Patient presents to hospital:

Slide 3:

A) Full exam room with the first patient in a bed next to a medical cart and supplies.

B) Click on the lower right box to play the video of the close up of the mannequin with bruises.

The facilitator announces that a family member has entered with this patient to give some medical history. The facilitator can play the role of the family member, giving specific details of the history only if the participants ask. While giving the history, A and B above are shown.

HPI: 24-year-old male is brought in by his family member. Two days ago he had a fever, headache, and body aches. Yesterday he felt worse, so he didn't go to work in their field or gathering wood. Today he started vomiting and had a red rash with some bruises. Now he is very uncomfortable.

Only if they ask: his wife didn't bring him in because she is at home with a headache and fever.

Review of Systems:

- Feeling well 3 days ago
- Started feeling worse yesterday
- Headache, fever, body aches
- Diarrhea started last night and continues today (doesn't know if it is bloody)
- Family member doesn't know of any other symptoms

Past Medical History:
Healthy
Never been in the hospital
No surgeries

Medications: None

Allergies: No known drug allergies

Social History: He is the oldest son. He lives in a family compound with his parents, wife, and 4 children. He works in farming planting coffee. They live near a dense forest where he frequently goes to gather wood. They live about 5 miles from the hospital in a rural area.

Other habits:
Food: Family grows their own food. They have 2 goats. They occasionally eat bush meat (i.e. meat of wild animals).
Water: They drink from a well that is shared between 5 family compounds.
Travel: He went to the capital city 1 year ago for a brief trip. Otherwise, he has not travelled outside the region.

Family History:
No chronic illnesses in family members.
His wife at home is sick with the symptoms he had yesterday.

Click "next" to advance the slide. Slide 4 will show.

Supplies shown in detail:

Slide 4: Close up of the equipment available

The facilitator reviews the available supplies and asks what the participants would like to do now. A medical cart is in the corner with:

-oxygen nasal cannula and mask
-suction catheter
-peripheral IV start supplies

-IV fluids with IV pole
-supplies for blood draw
-surgical masks
-non-sterile gloves
-yellow gowns
-stethoscope
-blood pressure cuff
-dextrose
-antimicrobials (including anti-malarial treatments)
-epinephrine
-atropine

Click “next” to advance the slide. Slide 5 will show.

Physical exam of first patient:

Slide 5:
A) First patient in bed with initial vital signs shown on monitor.
B) Audio clip of fine crackles and tachycardia (click on heart and lungs)

The facilitator shows this slide only when the participants ask for vitals and a physical exam. The facilitator gives the participants details of what they see/hear on the physical exam and click on the heart and lungs to play the fine crackles and tachycardia. The facilitator only gives a few minutes after reviewing the exam for initial actions before clicking on one of the 3 options in the lower right.

VS: **T: 40 deg C P: 140 (thready) R: 34 BP: 70/25 SPO2: 98%**
Exam: General: Somnolent, minimally responsive
Skin: Dehydrated (tenting, doughy)
HEENT: Normal (no dried blood around mouth, nose)
Respiratory: Fine crackles bilaterally
Cardiovascular: Tachycardic, regular
Abdomen: Soft, hypoactive bowel sounds
Extremities: Cool to touch
Neuro: Stuporous, moans to deep stimulation

Click “Back to supplies” to review available supplies. Slide 4 will be shown.

Click “Yes” if the participants have begun to resuscitate the first patient. This would include starting an IV and giving fluids, giving antimicrobials, sending blood for labs, and giving supplemental oxygen. Slide 6 will be shown.

Click “No” if the participants have not begun to resuscitate the first patient. Slide 7 will be shown.

Participants have begun to resuscitate the patient, but he begins to vomit:

Slide 6: First patient in bed with vital signs shown. IV with tubing is inserted and attached to a hanging IV fluid bag A nasal cannula/mask is in place. There is bloody emesis in a basin.

The vital signs are unchanged, as resuscitation has only just begun. The facilitator allows the participants to continue to talk about which steps they will take next in this scenario. In addition

to resuscitation with IV fluids, participants should check a point of care glucose when they send labs. They should give broad spectrum antibiotics and check a rapid malaria test. Labs that are drawn will have results come back about 15 minutes in to the scenario. The lab machines could be broken or reagents could be lacking if the facilitator would like to give less information (in which case they should administer glucose and anti-malarial medications).
Click "Next" after the participants have resuscitated the patient. Slide 8 will be shown.

Participants have not yet begun to resuscitate the patient, and he begins to vomit:

Slide 7: First patient in bed with vital signs shown. There is NO IV tubing/fluids/oxygen. There is bloody emesis in a basin.

The vital signs are unchanged. The facilitator allows the participants to talk about which steps they will take next in this scenario. The facilitator can give prompting if needed to be sure resuscitation is initiated (see slide 6 for details of resuscitation).
Click "Next" just as the participants begin to resuscitate the patient. Slide 6 will be shown.

Improved vitals after resuscitation of first patient:

Slide 8: First patient in bed with new vital signs and IV tubing attached to IV fluid bag and nasal cannula/mask in place.

Improved vital signs are now shown on the monitor.
Temperature: 40 degrees C, Pulse:100, Respiratory Rate: 25, Blood Pressure: 90/60 SPO2: 98%
Click "Next" approximately 10 minutes in to the scenario, regardless of participant discussion. Slide 9 will be shown.

About 10 minutes in, a second patient arrives:

Slide 9: Second patient in bed behind first patient with same medical cart in corner.

The facilitator announces the arrival of a second, younger patient in a second bed behind the first patient. A family member drops off this patient and leaves, so the facilitator can't give any additional history. The facilitator asks how the participants would like to proceed. The participants can finish briefly discussing resuscitation of the first patient, but they should begin to assess the second patient at this time.
Click "Next" when the participants ask for vital signs and an exam. Slide 10 will be shown.

Physical exam of second patient:

Slide 10:
A) Second patient in bed near first with monitor at bedside. Vital signs are shown on the monitor, and a medical cart is in the corner.
B) Audio clip of fine crackles and tachycardia (click on heart and lungs).

The facilitator shows this slide only when the participants ask for vitals and a physical exam. The facilitator gives the participants details of what they see/hear on the physical exam and click on the heart and lungs to play the fine crackles and tachycardia. The facilitator can not give any additional history for this patient.

VS: **T: 39 deg C P: 130 (thready) R: 30 BP: 75/30 SPO2: 98%**
Exam: General: unresponsive
Skin: Dehydrated (tenting, doughy), extensive bruising
HEENT: Dried blood around mouth and nose
Respieratory: Fine crackles bilaterally
Cardiovascular: Tachycardic, regular
Abdomen: Soft, hypoactive bowel sounds
Extremities: Cool to touch

Click on “Next” only after participants have resuscitated the second patient. Slide 11 will be shown.

Improved vitals after resuscitation of second patient:

Slide 11: First patient present in front, but second patient in bed with new vital signs and IV tubing attached to IV fluid bag and nasal cannula/mask in place.

Improved vital signs are now shown on the monitor.

Temperature: 38 degrees C, Pulse: 100, Respiratory Rate: 27, Blood Pressure: 95/55 SPO2 98%

Labs for the second patient should be drawn/sent, but results don’t return during this scenario.

If labs are drawn/sent for the first patient, the results will come back after about 15 minutes. The facilitator can announce the results at that time or wait until the participants ask again.

CBC: Hgb 6, WBC 2.1, Platelets 89
BMP: Na 133, K 3.9, Cl 105, CO2 16, BUN 29, Creat 2.2, Glucose 56
UA: large blood, otherwise unremarkable.
Malaria rapid diagnostics: Negative

This ends Day One.

Click on “Next” 15 minutes into the scenario. Slide 12 will show.

Day 2:

Slide 12: “Day Two”

The facilitator announces that time has passed and it is now the next day.

Click on “Yes” if both patients have been resuscitated. Slide 14 will show.

Click on “No” if both patients were not resuscitated. Slide 13 will show.

Both patients were resuscitated:

Slide 14: Both patients in beds near each other with IV tubing attached to IV fluid bag and nasal cannula/mask in place, both attached to monitors with improved vital signs.

Vital signs for both patients show on the monitors.

Temperature: 37 degrees C, Pulse: 105, Respiratory Rate: 27, Blood Pressure: 95/75, SPO2 98%

Temperature: 37 degrees C, Pulse: 100, Respiratory Rate: 25, Blood Pressure: 90/60, SPO2 98%

Click on “Next” after everyone has read the new vital signs. Slide 15 will show.

Both patients were not resuscitated:

Slide 13: Both patients in beds near each other with NO IV tubing attached to IV fluid bag and nasal cannula/mask in place, both attached to monitors with worsened vital signs.

Vital signs for both patients show on the monitors.

Temperature: 40 degrees C, Pulse: 130 (thready), Respiratory Rate: 32, Blood Pressure: 60/25, SPO2: 98%

Temperature: 39 degrees C, Pulse: 135 (thready), Respiratory Rate: 36, Blood Pressure: 65/30, SPO2: 98%

If the group has not done basic resuscitation steps (IV fluids, antibiotics, etc.), the facilitator briefly prompts the group to resuscitate both patients at this time.

Click on “Next” after everyone has read the new vital signs. Slide 14 will show.

Hospital administrator arrives:

Slide 15: “Hospital Administrator arrives”

The facilitator announces that the hospital administrator arrives on Day 2 to speak to the medical team (participants). The facilitator can play the role of the hospital administrator. If the team asked for the hospital administrator before, the facilitator says someone has left to fetch them. The hospital administrator will have the following conversation, and hint that a village assessment should be considered if the medical team doesn't think of it.

The hospital administrator reports that a nurse just said a father and daughter came in and died. The hospital cleaner saw the blood and heard the story, and she has refused to return to work as it is dangerous. Nurses have been staying home for the same reason. The hospital administrator asks what is happening and what should be done.

- If the medical team says that they know this is an outbreak, the hospital administrator does not know what to do and tells the team to please figure out what to do.
- The administrator asks about collaboration with the town council and local NGOs, who have the following particular questions:
  - What changes have to happen in the hospital during this outbreak?
  - Are there any changes that have to happen in the community?
  - Who is most at risk?
  - What should they advise people at risk to do?
  - What should they tell the community and how should they reach them?

Click "Next" when the participants ask for a team to be sent to the homes of the patients' village for an assessment. Slide 16 will be shown.

House in a rural village near Makeni, Sierra Leone:
Slide 16: Inside a house with an adult Manikin and dead child Manikin, both with evidence of hemorrhage. Both represent dead patients.
The facilitator reports that there several houses with very sick people or those who have just died in the village.
Click "Next" after reporting this assessment and after any responses from the participants. This should be 1-2 minutes before the end of the case.

Outbreak unfolding in hospital:
Slide 17: More Manikins in the same hospital ward, with evidence of hemorrhage. Click to play video of the hospital ward.
The facilitator notes that this is evidence of an unfolding outbreak. If a differential diagnosis hasn't been discussed and narrowed by this time, the facilitator asks pointed questions to help the participants come to some conclusions. The patients had Ebola Virus Disease, and they should be managed as if they had any hemorrhagic fever, with resuscitation, searching for comorbidities/other items on the differential diagnosis that need specific treatments, with maximal isolation precautions, and with consideration to containment and effects on the community. Participants should also be communicating and collaborating with local partners as well as seeking help from the outside.
Stop sharing your screen and end the slideshow and scenario here.

Debriefing Objectives:

The facilitator concludes the case after 20 minutes to allow for debriefing. Debriefing begins with questions/comments/feedback from participants regarding the case, the steps they took, and working together. Then, the group can discuss the following:

1) Discuss how you work-up and treat a hemorrhagic fever in a low-resource setting (plus, background on EVD)
2) Discuss how you handle an outbreak of a public health emergency in a low-resource setting (case management, infection prevention and control practices, surveillance and contact tracing, safe lab practices, safe and dignified burials, and social mobilization).
3) Discuss resources for help
4) Discuss how you set up a community to be prepared for future outbreaks

Critical Actions:

1) Objective 1
   a. Gather information from clinical history and exam, and consider epidemiologic information (risk of exposure) to classify the patient as a person under investigation.
   b. Initiate standard precautions (always) then droplet/contact precautions.
   c. Isolate patient(s).
   d. Collect blood (or saliva if blood is not possible) sample to send for analysis.
   e. Create a triage system in the hospital: isolation of sick contacts, EVD RT-PCR + patients. Recognize the need for a safe lab and safe, dignified burials.

2) Objective 2
   a. Attend to A, B, C's.
   b. Recognize that this is a viral hemorrhagic fever. Give supportive treatment (fever/pain, IV fluids, consider giving antibodies if appropriate).
      i. glucose, empiric treatment of possible co-infections

3) Objective 3
   a. Contact local and national health care authorities. Request additional human resources.
   b. Assess resources and communicate needs to local and national health authorities.
   c. Create a plan for surveillance and contact tracing, safe transport of symptomatic people.
   d. Quarantine contacts.
   e. Begin to plan social mobilization, balancing fear and chaos to reduce stigmatization.

4) Objective 4
   a. Recognize the possibility of using monoclonal antibody or vaccines for EVD.

EVD Handout

Ebola Virus Outbreaks by Species and Size, Since 1976

WHO resource: https://www.who.int/news-room/fact-sheets/detail/ebola-virus-disease
https://www.cdc.gov/vhf/ebola/history/distribution-map.html

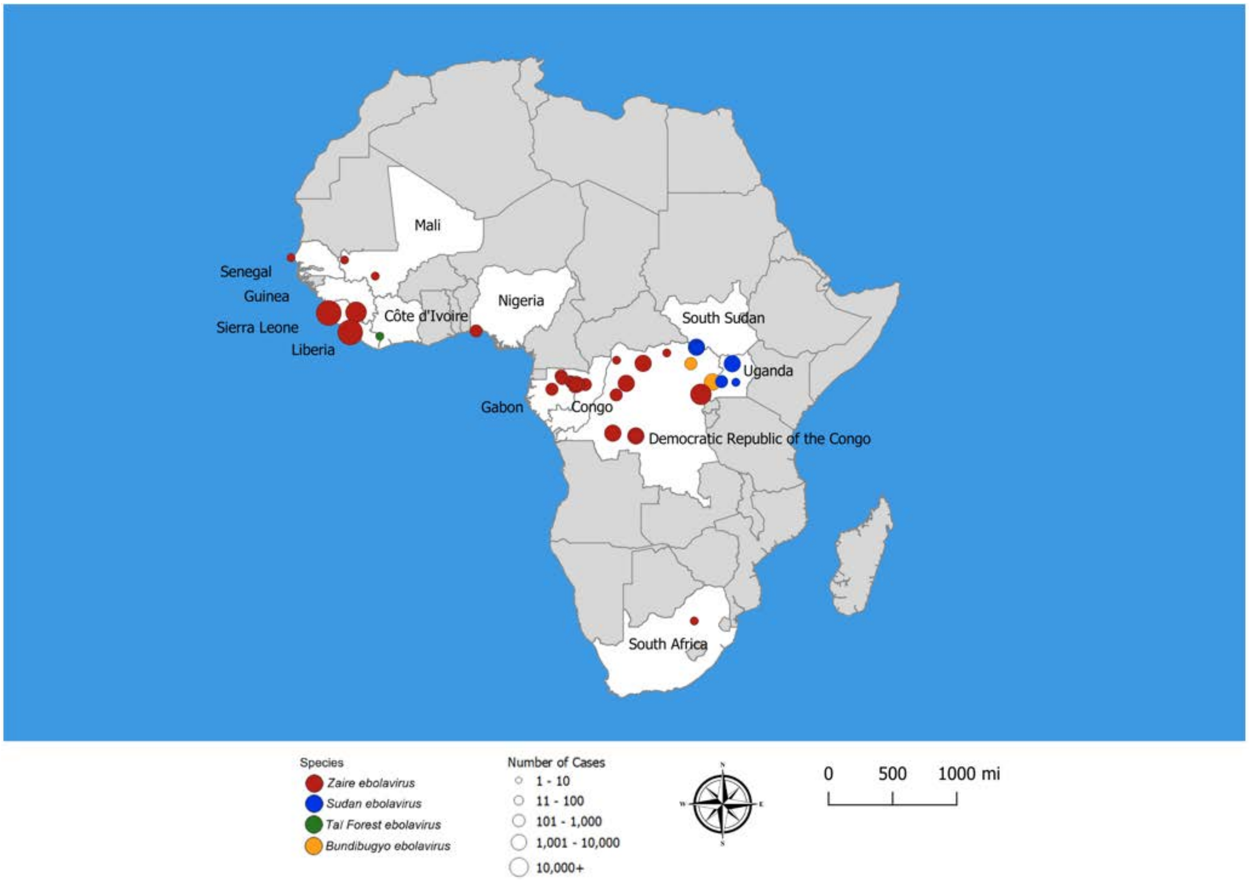

The Ebola virus can cause severe viral hemorrhagic fever (VHF) outbreaks in humans with an average case fatality rate of up to 90%. With fruit bats thought to be its natural hosts, Ebola is introduced into the human population through close contact with the blood, secretions, organs or other bodily fluids of infected animals. In Africa, infection has been documented through the handling of infected chimpanzees, gorillas, fruit bats, monkeys, forest antelope and porcupines found dead or ill in the rainforest.

Later Ebola spreads in the community through human-to-human transmission, resulting from close contact with the blood, secretions, organs or other bodily fluids of infected people. Burial ceremonies where mourners have direct contact with the body of the deceased person can also play a role in the transmission of Ebola. Transmission via infected semen can occur >9 months after clinical recovery.

Health-care workers have frequently been infected while treating Ebola patients. This has occurred through close contact without the use of correct infection control precautions and adequate barrier nursing procedures. For example, healthcare workers not wearing gloves and/or

masks and/or goggles may be exposed to direct contact with infected patients' blood and are at risk.

EVD (Ebola virus disease) is a severe acute viral illness often characterized by the sudden onset of fever, intense weakness, myalgias, headache, and sore throat. This is followed by vomiting, diarrhea, rash, impaired renal and hepatic function, and in some cases, both internal and external bleeding. Laboratory findings show low counts of white blood cells and platelets as well as elevated liver enzymes.

The incubation period (interval from infection to onset of symptoms) varies between 2 to 21 days. A person is infectious at the time symptoms appear, and they remain infectious as long as their blood or secretions contain the virus. During EVD outbreaks, the average case-fatality rate is around 50% (has varied in past outbreaks from 25% to 90%).

Ebola virus infections can only be diagnosed definitively in the laboratory by a number of different tests:

- Automated/semi-automated nucleic acid tests (NATs) for routine diagnostic management
- enzyme-linked immunosorbent assay (ELISA)
- antigen detection tests (rapid antigen detection tests are used in remote settings where NATs aren't readily available, still confirm diagnosis with NATs)
- serum neutralization test
- reverse transcriptase polymerase chain reaction (RT-PCR) assay
- electron microscopy
- virus isolation by cell culture

Early supportive care with rehydration and symptomatic treatment improves survival, and severe cases require intensive supportive care. Patients are frequently dehydrated and in need of intravenous fluids or oral rehydration with solutions containing electrolytes. Co-infections should also be considered and treated empirically. Two monoclonal antibodies (Inmazeb and Ebanga) have been approved for infection in adults and children by US FDA (as of late 2020).There is no licensed treatment for EVD, but many blood, immunologic, and drug therapies are under development and are used in outbreaks.

Protective against the Zaire ebolavirus, the Ervebo vaccine is recommended by the Strategic Advisory Group of Experts on Immunization (safe and effective). In 12/2020, this vaccine was approved by US FDA and prequalified by WHO for people 18 yrs and older (excluding pregnant and breastfeeding women). In the 2018-2020 outbreaks in the DRC and in Guinea, it has been given to > 350,000 people under compassionate use protocol. A global stockpile of the Ervebo vaccine has been available since 1/2021: Zabdeno is the first dose, and the second dose, Mvabea, is given 8 wks later. This is a prophylactic 2 dose regimen that will not give immediate protection as is needed in outbreak response.

EVD can persist in some survivors in the following places: testicles, inside the eye, CNS, placenta, amniotic fluid, fetus if infected when pregnant, and breastmilk (if infected when breastfeeding). There is viral persistence (RT-PCR) in a small percentage of survivors more than 9 months after infection. Relapse of symptomatic illness is rare. EVD survivors are recommended to have no or safe sex for 12 months from onset of symptoms or until semen has tested negative twice. Survivors and their sex partners should also practice good hand and personal hygiene immediately after contact with body fluids. Routinely, males should have semen testing at 3 months after onset of disease and then every month until they have 2 negative tests by RT-PCR (1 wk between tests).

Community engagement is essential to outbreak control: case management, infection prevention and control practices, surveillance and contact tracing, good lab service, safe and dignified burials, and social mobilization.

Case management: Isolated, supportive treatment with barrier nursing precautions. Severely ill patients are treated in intensive care, separated from other patients, with EVD contacts kept in isolation for 21 days of observation.

Infection prevention and control practices: Will need coordination of prevention and control activities and resource mobilization (including prevention of contact with patient's blood and body fluids and contaminated surfaces, clothing, and bedding.) Healthcare setting precautions: face protection, clean long-sleeved gown, gloves. Separate healthy from sick, maintain good hygiene in a clean environment. Organize safe transport from patient's home to isolated healthcare area.

Surveillance and contact tracing: Surveillance of community for human EVD cases as well as surveillance of pig farms (potential sites of virus amplification). Strengthen food production system, prevent bats from introducing Ebola into pig populations, and contain infection in pig populations. Monitor health of EVD contacts for 21 days, and refer to an isolation ward if they become ill.

Lab precautions: extreme biohazard risk (biosafety level 4), needs maximum biological containment (triple packaging system)

Safe and dignified burials, social mobilization, and community engagement: awareness of risk factors and protective measures (hygiene, vaccination)

Risk reduction messages:

1- Reduce risk of wildlife to human transmission (infected bats, primates, consumption of raw meat) by using gloves and cooking meat thoroughly.

2- Reduce risk of human to human transmission by using gloves and personal protective equipment (PPE) when bodily fluid contact when caring for infected patients.

3- Reduce risk of sexual transmission by practicing abstinence or safe sex (and wash with soap/water if in contact with semen) for 12 months from symptom onset or until semen tests negative twice.

WHO's strategy for the prevention and control of EVD outbreaks:

Phase 1: Pre-epidemic preparedness: A surveillance system can identify cases in animals and/or humans that would trigger implementation of a prevention program. Always use standard precautions with body fluids and potentially contaminated environment. Educate the public about potential risk behaviors, targeting hunters (importance of hand washing and handling raw meat), and burial groups.

Phase 2: Alert: Immediately investigate and evaluate risks, with a team (with PPE) sent to the site to evaluate risk of outbreak, carefully collect specimens to be sent to national reference lab, and implement initial control measures until lab results return. Assess local resources and needs, and make a decision after the lab results return.

Phase 3: Epidemic: Implement outbreak response and containment operations, with safe lab practices, infection control precautions, barrier nursing procedures, PPE, disinfection of contaminated objects/areas, and safe burials. Psychosocial support for healthcare workers, patients, families, and communities.

Phase 4: Post-epidemic evaluation: Resume surveillance activities from pre-epidemic phase, official announcement to thank stakeholders and extend solidarity and compassion (reduce stigma), make report of evaluation of outbreak management, and maintain records for international reference. Psychosocial support for healthcare workers, patients, families, and communities.

References:

Ebola and Marburg virus disease epidemics: preparedness, alert, control and evaluation, August 2014, WHO.
https://apps.who.int/iris/bitstream/handle/10665/130160/WHO_HSE_PED_CED_2014.05_eng.pdf;jsessionid=9BA643E86D309D9467F916BEAA9131FB?sequence=1

https://www.who.int/news-room/fact-sheets/detail/ebola-virus-disease

https://www.cdc.gov/vhf/ebola/index.html

Virtual Simulation

Next

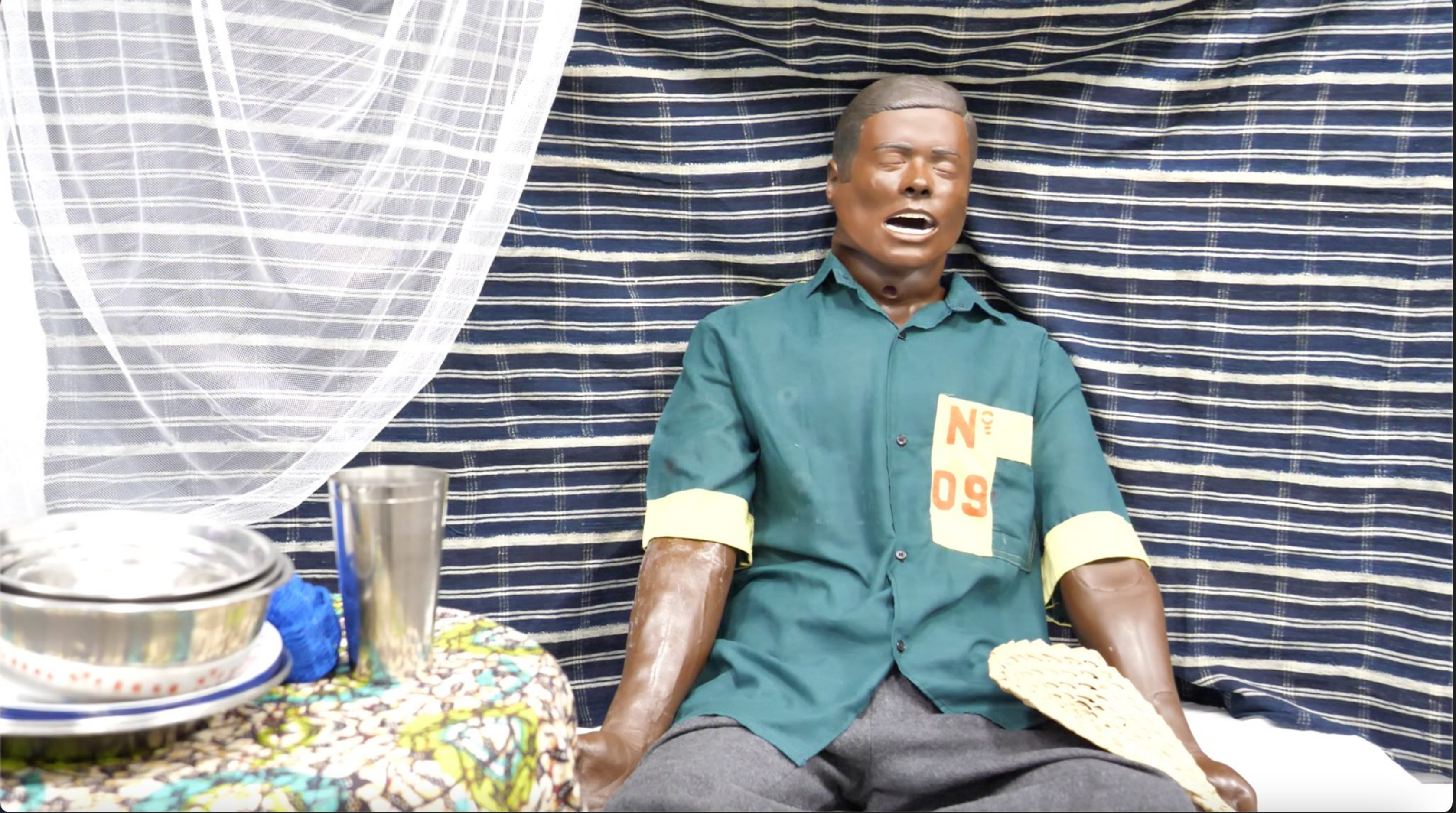

Village in Sierra Leone

Next

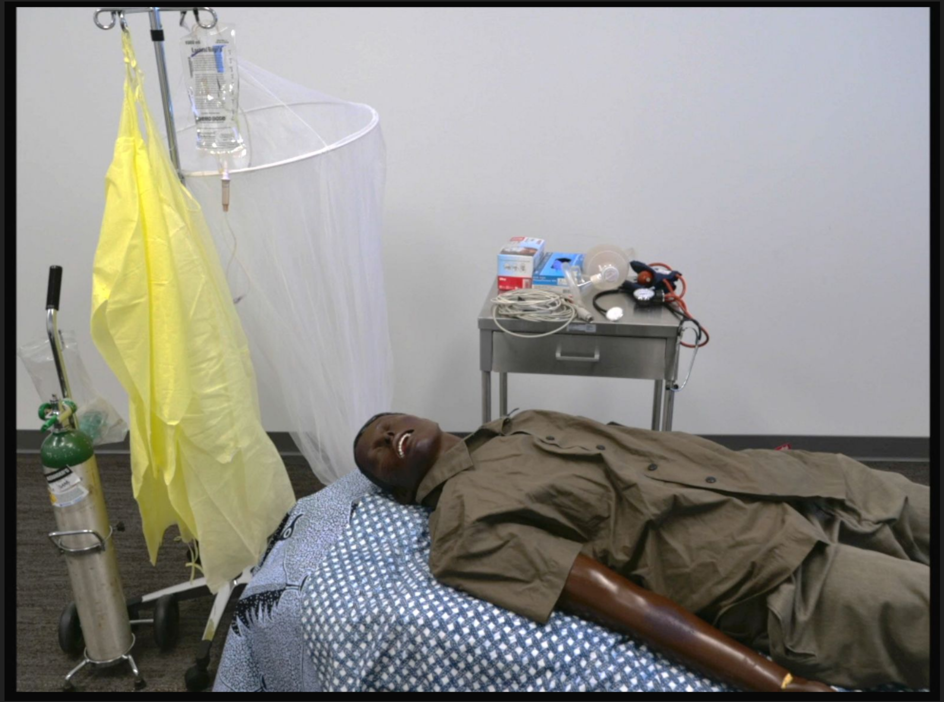

Rural Hospital in Sierra Leone

Next

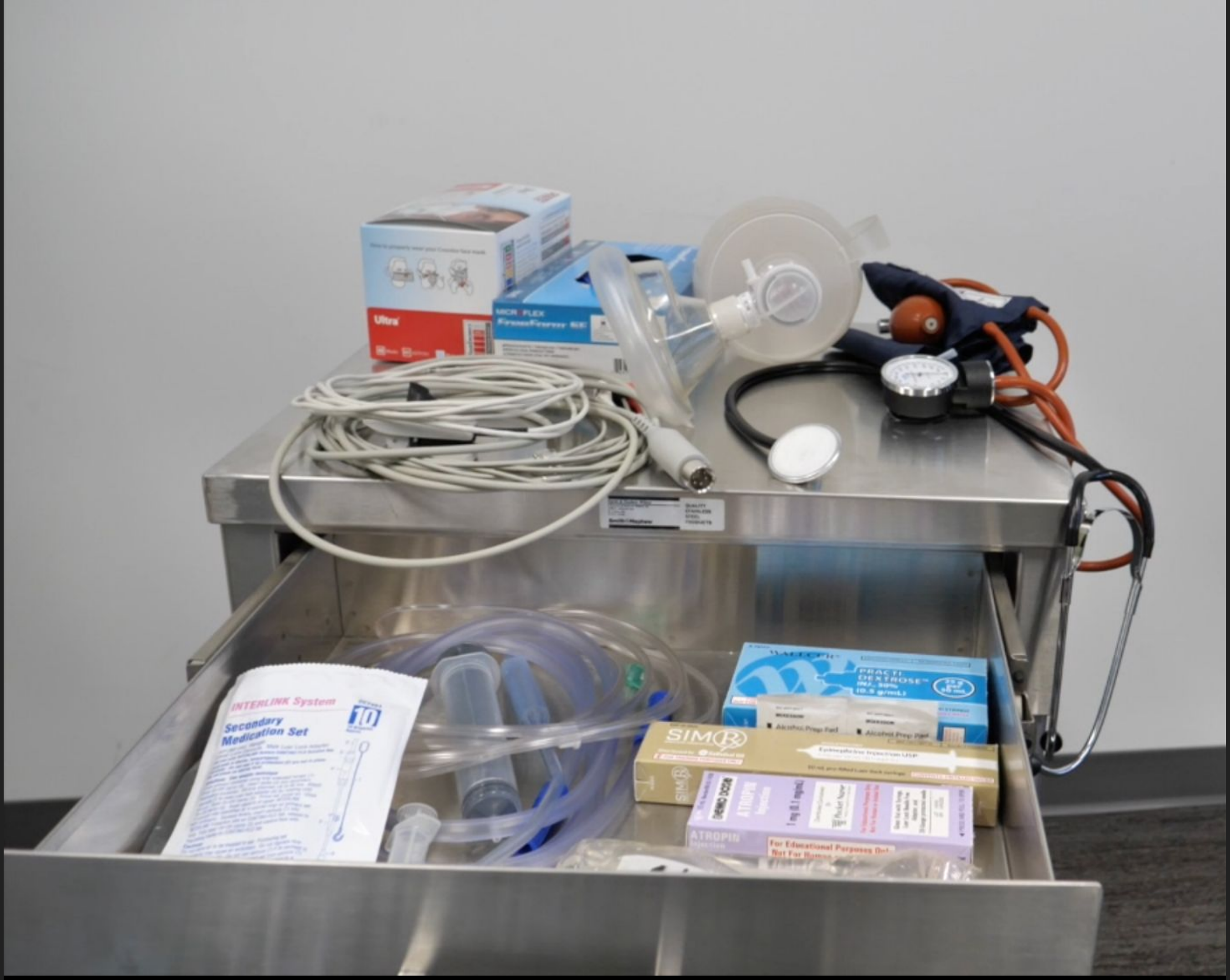

- oxygen nasal cannula and mask
- suction catheter
- peripheral IV start supplies
- IV fluids
- blood draw supplies
- surgical masks
- non-sterile gloves
- yellow gowns
- stethoscope
- blood pressure cuff
- dextrose
- antimicrobials
- epinephrine
- atropine

Next

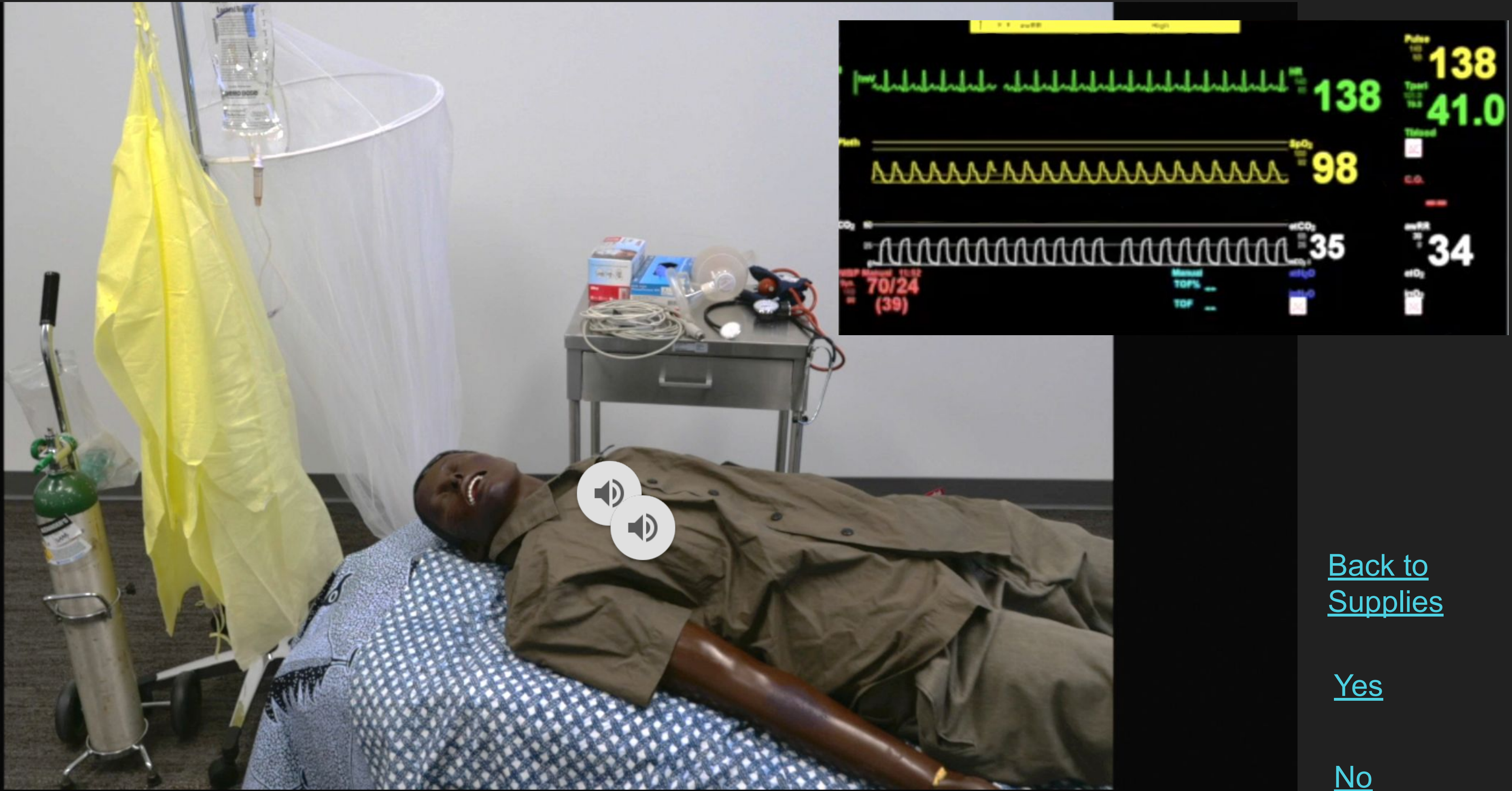

Back to Supplies

Yes

No

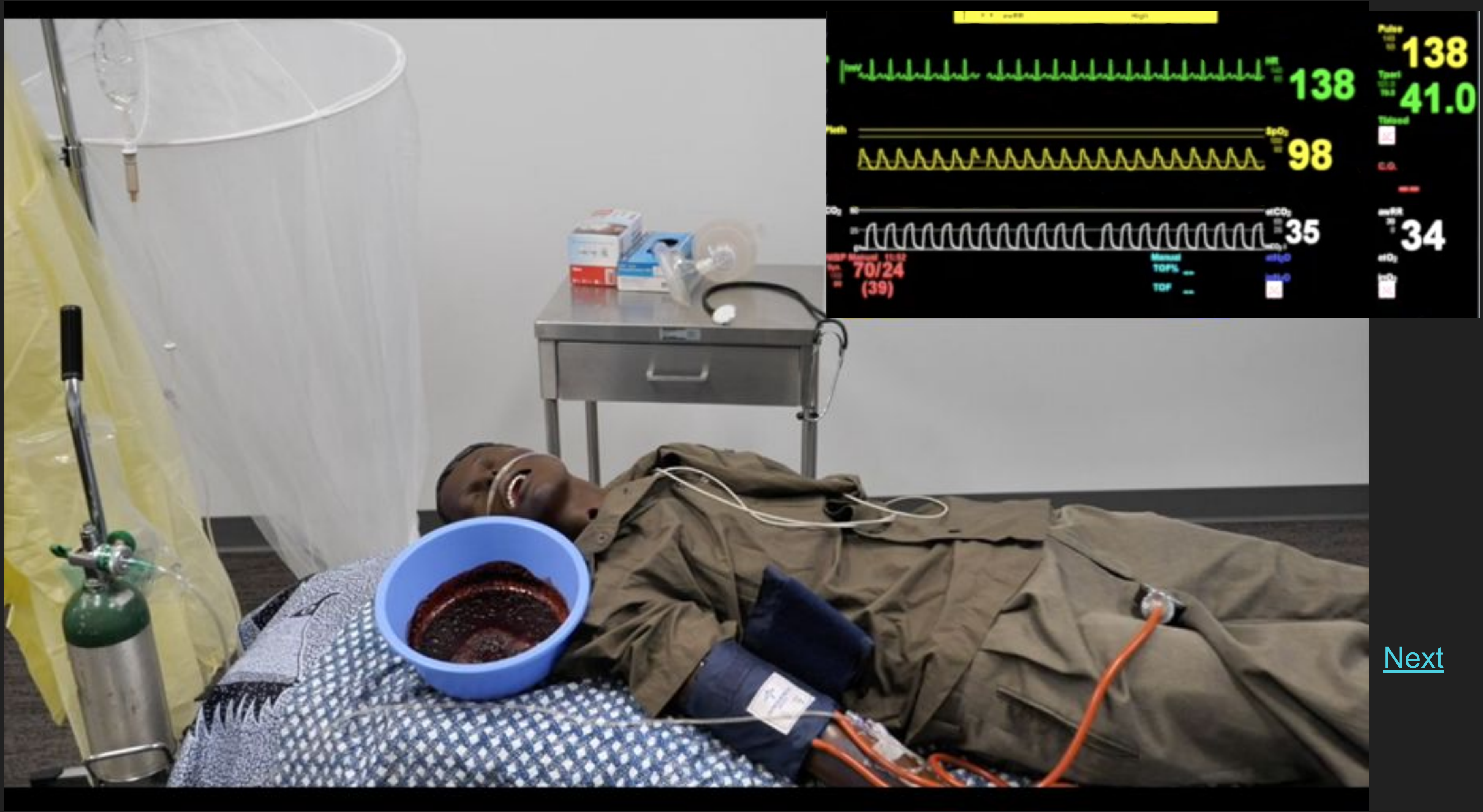

Next

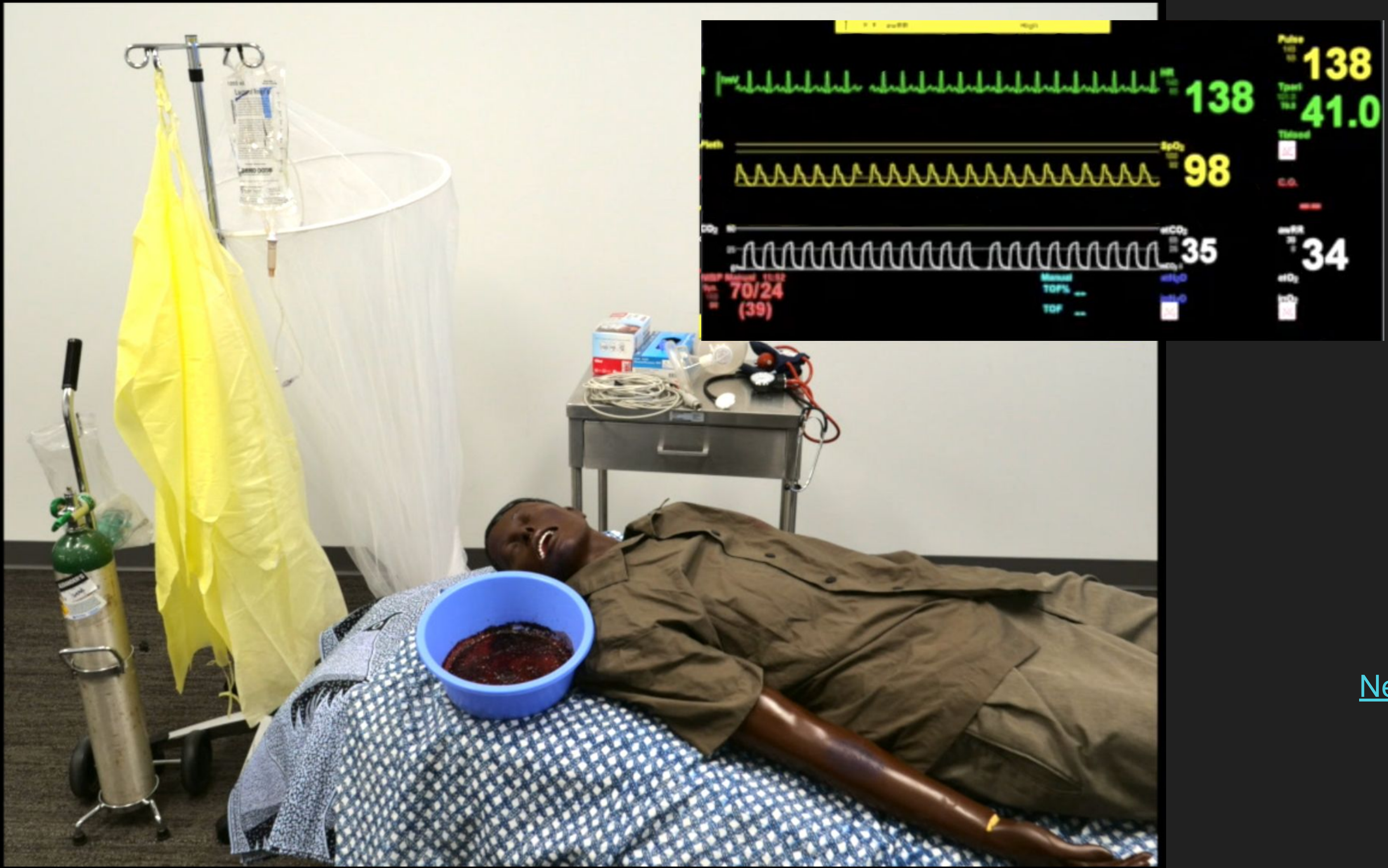

Next

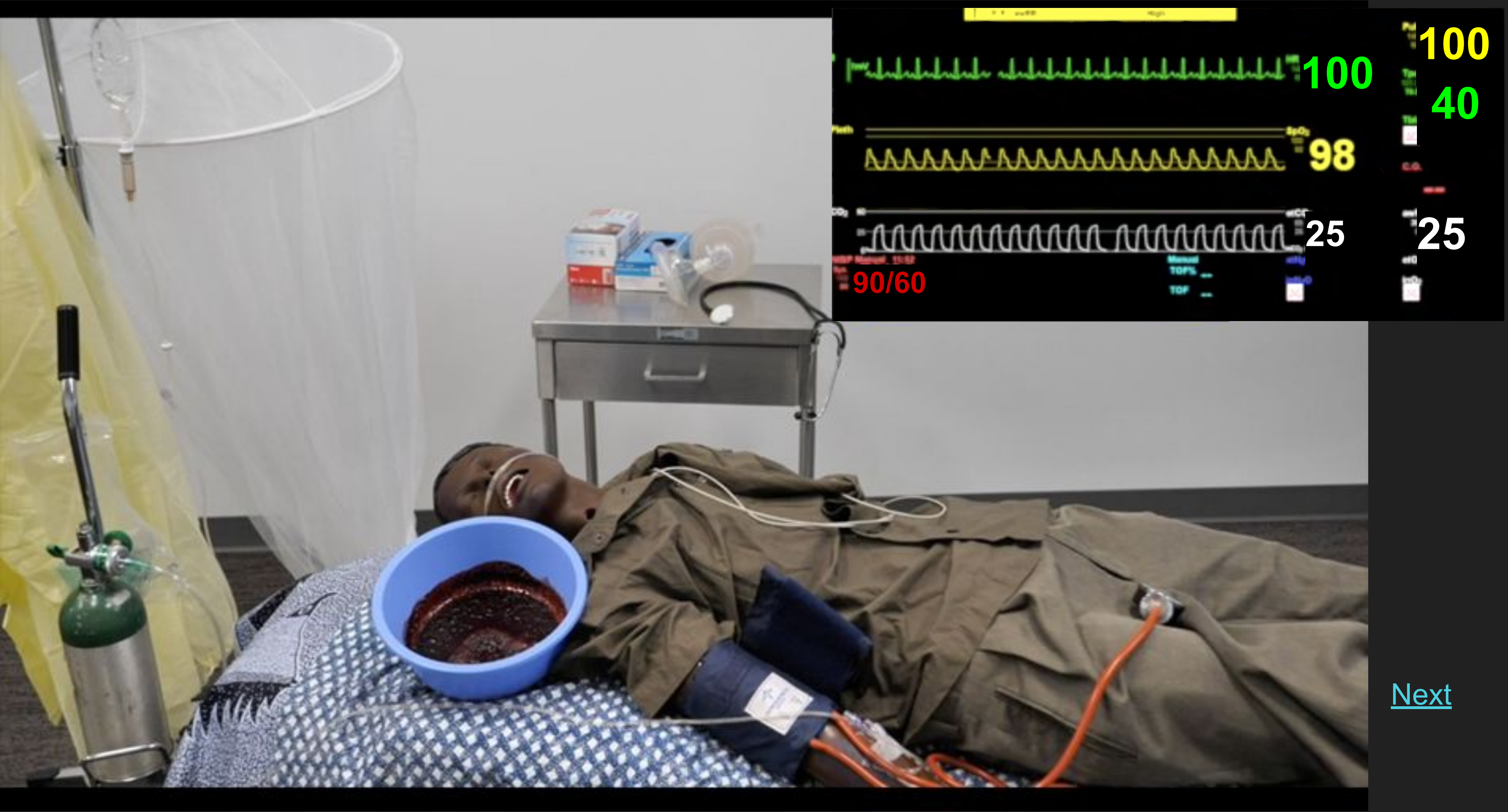

Next

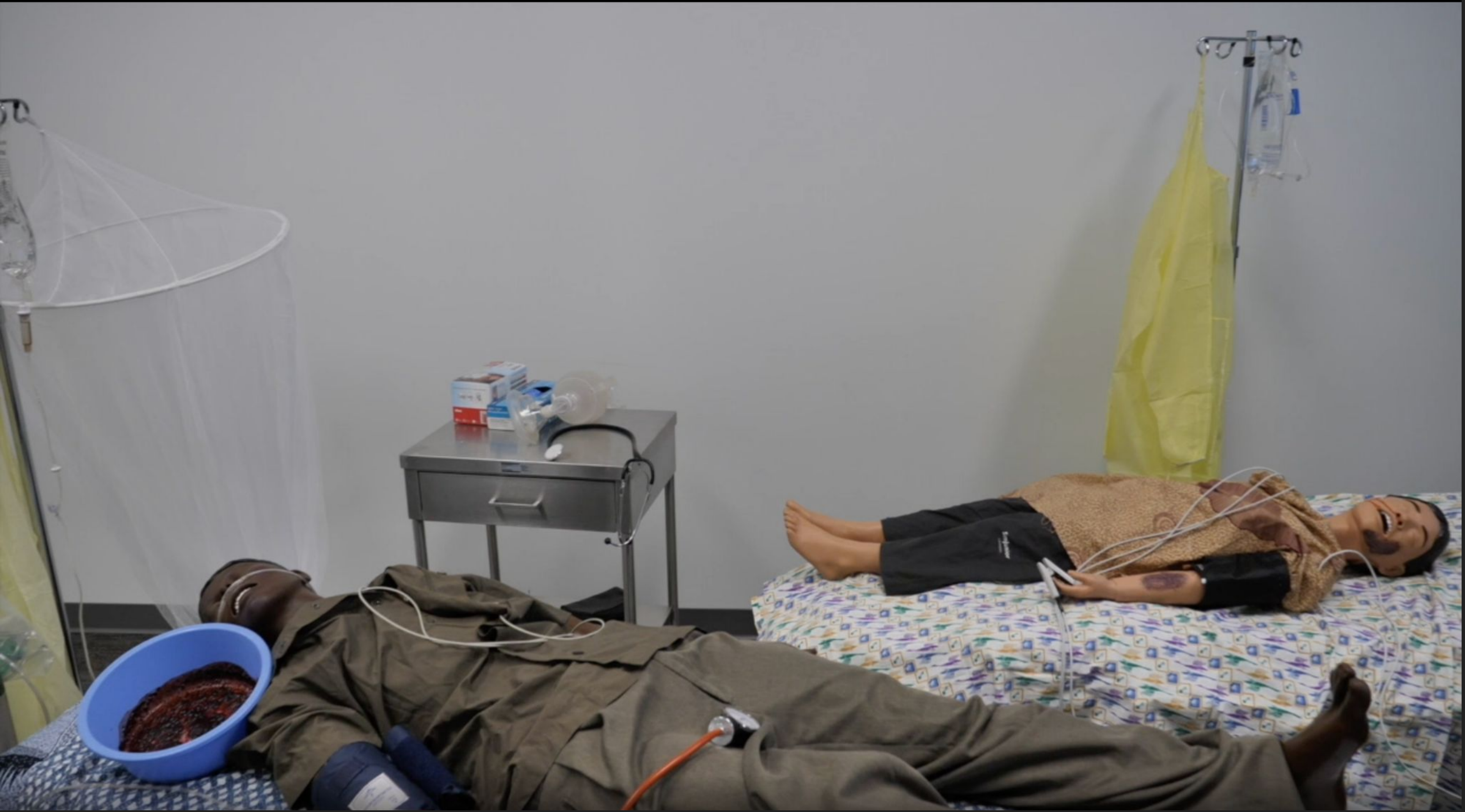

Next

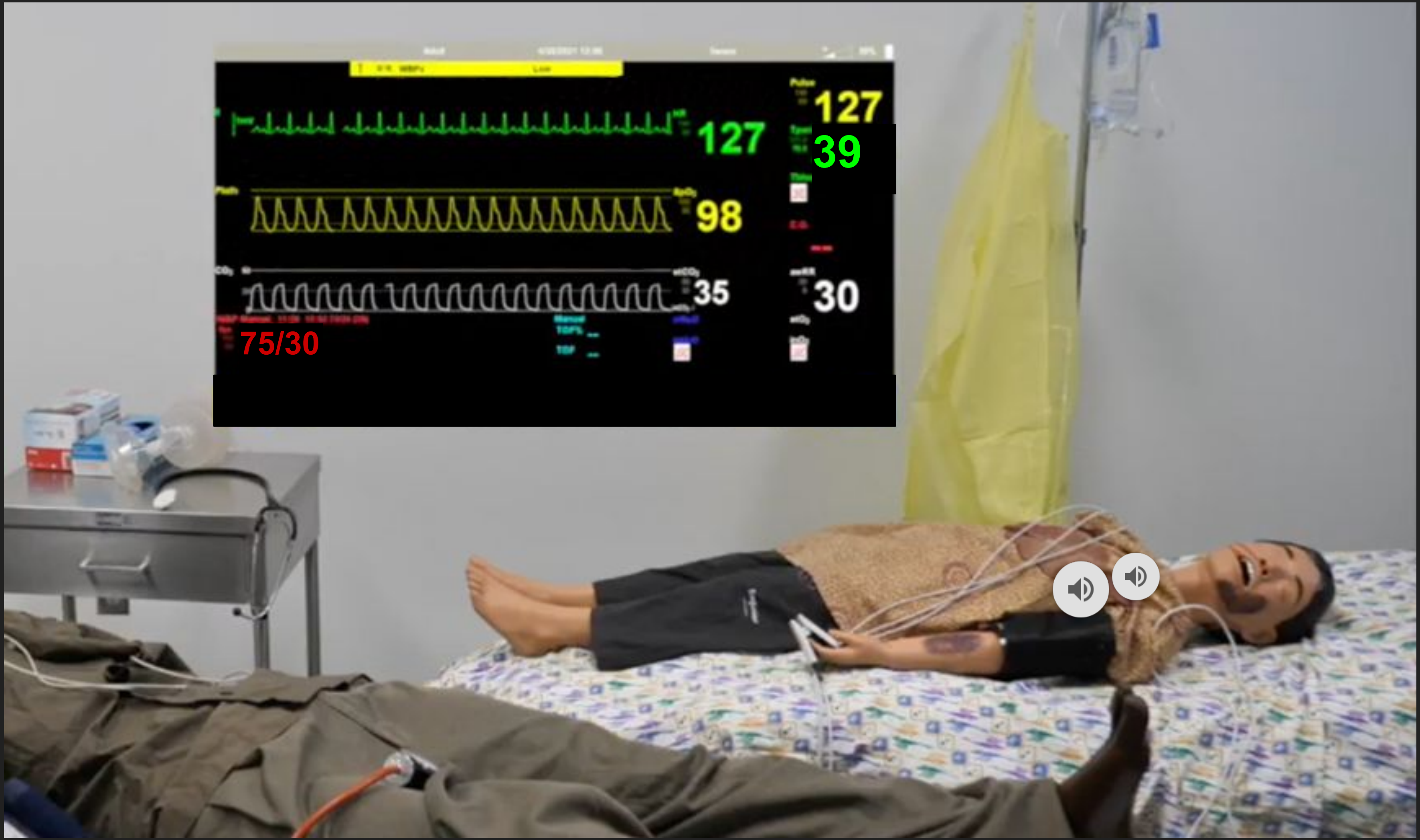

Next

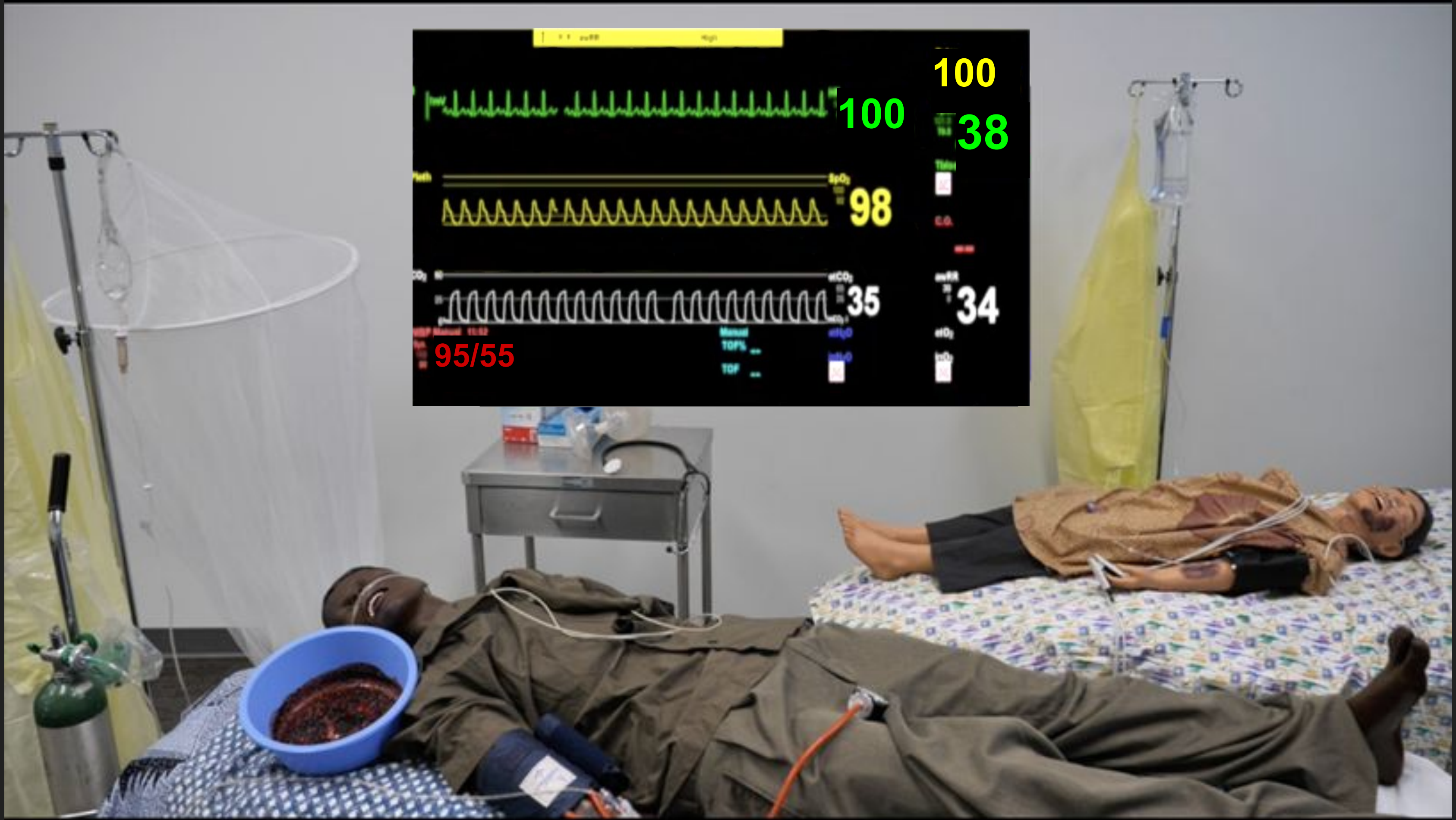

Next

DAY 2

yes

no

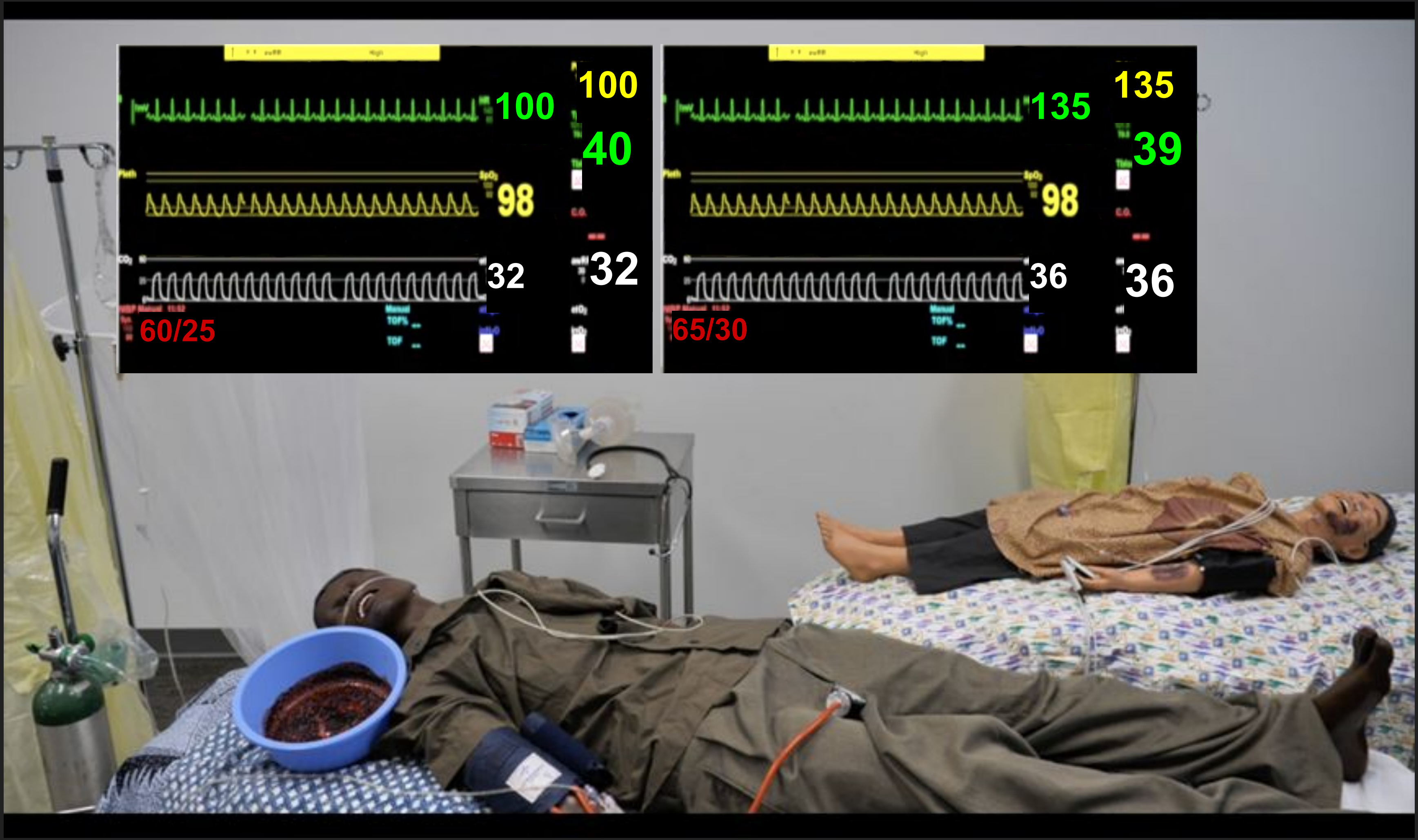

Next

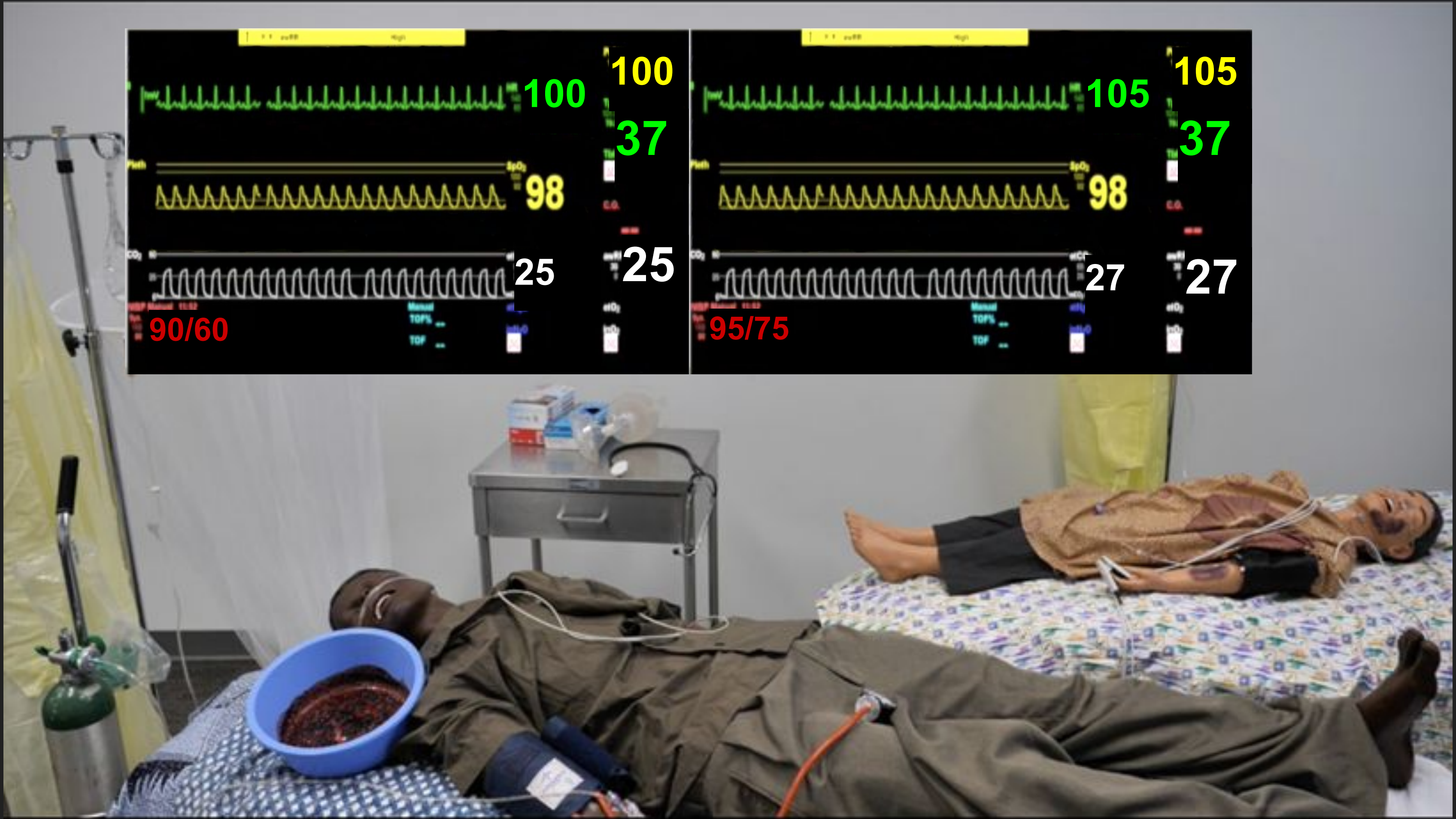

Next

Hospital Administrator arrives

Next

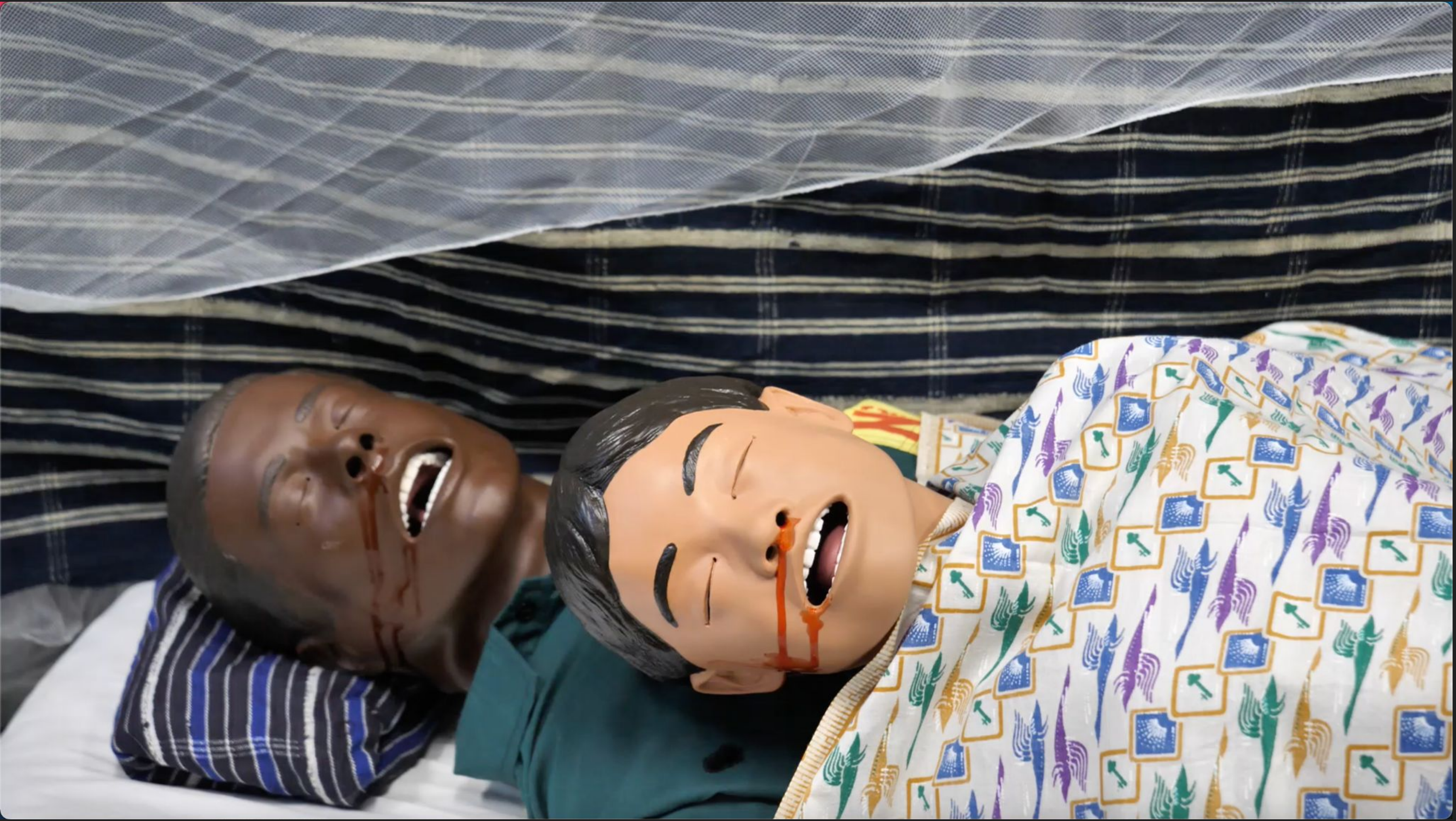

Next
